# Prognostic changes after sudden deafness in patients with inner ear malformations characterized by LSCC: a retrospective study

**DOI:** 10.3389/fneur.2023.1174412

**Published:** 2023-06-02

**Authors:** Lei Chen, Qinglei Dai, Xin Gao, Na Hu, Xiao Sun, Haibo Wang, Mingming Wang

**Affiliations:** ^1^Department of Otolaryngology Head and Neck Surgery, Shandong Provincial ENT Hospital, Cheeloo College of Medicine, Shandong University, Jinan, China; ^2^Department of Otology Medicine, Shandong Provincial ENT Hospital, Shandong University, Jinan, China; ^3^Department of Radiology, Shandong Provincial ENT Hospital, Shandong University, Jinan, Shandong, China

**Keywords:** lateral semicircular canal malformation, semicircular canal, sudden sensorineural hearing loss, prognosis, vestibular function, dizziness

## Abstract

**Introduction:**

This study aimed to investigate the clinical features and prognosis of sudden sensorineural hearing loss in patients with lateral semicircular canal (LSCC) malformation.

**Methods:**

This study enrolled patients with LSCC malformation and sudden sensorineural hearing loss (SSNHL) who were admitted to Shandong ENT Hospital between 2020 and 2022. We collected and analyzed data on examinations of audiology, vestibular function, and imaging records of patients and summarized the clinical characteristics and prognosis of these patients.

**Results:**

Fourteen patients were enrolled. Patients with LSCC malformation was noted in 0.42% of all SSNHL cases during the same period. One patients had bilateral SSNHL and the rest had unilateral SSNHL. Of them, eight and six patients had unilateral and bilateral LSCC malformations, respectively. Flat hearing loss was noted in 12 ears (80.0%) and severe or profound hearing loss was noted in 10 ears (66.7%). After treatment, the total efficacy rate of SSNHL with LSCC malformation was 40.0%. Vestibular function was abnormal in all patients, but only five patients (35.7%) had dizziness. There were statistically significant differences in the vestibular functions between patients with LSCC malformation and matched patients without the malformation hospitalized during the same period (*p* < 0.05).

**Conclusion:**

Patients with SSNHL and LSCC malformation had flat-type and severe hearing loss and worse disease prognosis compared to those with SSNHL without LSCC malformation. Vestibular function is more likely to be abnormal; however, there was no significant difference in vestibular symptoms between patients with and without LSCC malformation. LSCC is a risk factor for the prognosis of SSNHL.

## 1. Introduction

Semicircular canal malformation is a rare type of inner ear malformation. In the fifth week of embryonic development, the superior semicircular canal develops, followed by the posterior semicircular canal and finally the lateral semicircular canal (LSCC), which is most prone to malformation. Therefore, malformations of the superior and posterior semicircular canals are always accompanied by LSCC abnormalities. Malformation of an isolated LSCC has been reported (1). LSCC malformation can occur with other inner ear malformations, including cochlear, vestibular, and vestibular aqueducts, depending on the stage of inner ear development (2). The malformed LSCC are usually short and wide but may be narrow. In extensive malformations, the vestibular is dilated and forms a common lumen with LSCC (3). The imaging diagnosis can be a temporal bone computed tomography (CT) finding in which the central bone island of the LSCC is shorter than 7 mm (4).

Sudden sensorineural hearing loss (SSNHL) is defined as a sudden, unexplained sensorineural hearing loss of ≥30 dB in at least three consecutive frequencies within 72 h (5). SSNHL can occur in patients of any age and does not vary with sex, side, season, or geographic area. Its clinical manifestations can be accompanied by vertigo or dizziness (6, 7). SSNHL can also occur in patients with LSCC malformation; however, few studies have examined the clinical characteristics of these patients. Thus, this study aimed to investigate the clinical features and prognosis of SSNHL in patients with LSCC malformation. To the best of our knowledge, no study has investigated this.

## 2. Materials and methods

### 2.1. Participants

A retrospective analysis was conducted on 14 patients with LSCC malformation who were hospitalized for SSNHL at Shandong ENT Hospital between 2020 and 2022. After admission, they underwent a detailed medical history inquiry, audiology examination, vestibular function examination, and imaging examination. We selected 165 matched patients admitted to the hospital during the same period as the control group. This study was conducted in accordance with the Declaration of Helsinki and was approved by committee ethics board of the hospital (No. 2023-006-01); informed consent was obtained from all the participants.

### 2.2. Inclusion and exclusion criteria

Patients presented with SSNHL, excluding retrocochlear diseases and middle ear lesions. Patients with LSCC malformation (with or without vestibular malformation) who underwent CT of their temporal bones, did not undergo treatment before admission, course of disease was less than 1 month, and had no contraindications with glucocorticoids. A total of 165 patients hospitalized during the same period and matched with clinical characteristics without LSCC deformity were selected as the control group.

### 2.3. Audiology examination

All patients underwent acoustic immittance (GSI TympStar, United States), pure-tone audiometric threshold tests (GSI-61, United States), distortion product optoacoustic emissions (IHS Smart EP, United States), auditory brainstem responses (IHS Smart EP, United States) before treatment to exclude other lesions. Pure-tone audiometric threshold tests were performed twice a week during treatment.

### 2.4. Vestibular function examination

All patients underwent video head impulse test (vHIT, Ulmer, SYNAPSIS, Marseille, France), caloric testing (Ulmer VNG, v. 1.4; SYNAPSYS, Marseille, France), vestibular autorotation test (VAT, Western Systems Research, Pasadena, United States) and vestibular-evoked myogenic potential testing (VEMP, Neurosoft Ltd., Ivanov, Russia). An abnormal test was considered to indicate abnormal vestibular function.

### 2.5. Imaging tests

Magnetic resonance imaging of the inner ear was performed in all patients to rule out abnormal development of the cochlea and inner auditory canals. Patients with abnormal vestibular and semicircular canals were diagnosed using high-resolution CT (HRCT) of the temporal bone.

### 2.6. Classification and treatment

According to German Guidelines (7), patients were classified into the following four groups. Total hearing loss group (affecting all frequencies with an average threshold ≥81 dB HL), flat-type group (affecting all frequencies with an average threshold ≤80 dB HL), high-frequency group (at least affecting frequencies of 4 and 8 kHz) and low-frequency SSNHL group (affecting frequencies of 250, 500, and 1,000 Hz). The degree of hearing loss was graded according to the pure-tone average (PTA) at the damaged frequencies: normal, ≤25 dB HL; mild, 26–40 dB HL; moderate, 41–60 dB HL; severe, 61–80 dB HL; and profound, ≥81 dB HL. According to the treatment protocol of the Chinese Guidelines for the Diagnosis and Treatment of Sudden Deafness (2015), all patients were treated after admission. We used glucocorticoids, neurotrophic drugs, and hemodynamic therapy to improve blood flow, blood thinning and viscosity reduction. Efficacy evaluation of treatment included complete recovery, the improvement of the affected ear within 10 dB HL of the hearing level of the unaffected ear or to normal; marked recovery, ≥30 dB HL improvement in PTA of the damaged frequencies; slight recovery, the PTA of damaged frequencies improved by15–30 dB HL improvement; and no recovery, the PTA of the damaged frequencies improved by <15 dB HL.

### 2.7. Statistical analysis

In this study, the chi-square test was used for all test methods, and Fisher’s precision probability test was used when the chi-square test could not be performed. SPSS 20.0 software was used for statistical analysis, and *p* < 0.05 indicated statistical significance.

## 3. Results

This study enrolled patients with SSNHL who were admitted to our hospital between January 2020 and December 2022. Of the 14 patients with LSCC malformation confirmed using HRCT of the temporal bone, 13 patients had unilateral SSNHL and one patient had bilateral SSNHL. The average age of the patients was 46.0 ± 12.3 years and 13 patients were male. Eight patients had unilateral LSCC malformation (Tables 1, 2), and six had bilateral LSCC malformation (Tables 3, 4). Four of the eight patients with unilateral LSCC malformation were associated with vestibular malformation, three of the six patients with bilateral malformation were associated with bilateral vestibular malformation, and none of these patients had cochlear malformation. Patients with LSCC malformation accounted for 0.42% of the patients with SSNHL during the same period.

**Table 1:** Clinical characteristics of unilateral LSCC malformation patients with SSNHL.

Patient No.	Sex	Age	Side of LSCC malformation	Combined vestibular malformation	Side of SSNHL	Degree of hearing loss	Efficacy of treatment
1	M	57	L	No	L	L: Severe	I
						R: Normal	
2	M	43	L	No	L	L: Profound	III
						R: Mild	
3	M	33	R	No	L	L: Mild	IV
						R: Normal	
4	M	38	L	No	R	L: Profound	IV
						R: Moderate	
5	M	54	L	Yes	L	L: Severe	IV
						R: Normal	
6	M	52	R	Yes	L	L: Severe	I
						R: Normal	
7	M	52	R	Yes	L	L: Severe	IV
						R: Profound	
8	F	58	R	Yes	L	L: Severe	IV
						R: Normal	

M, male; F, female; L, left; R, right; I, complete recovery; II, marked recovery; III, slight recovery; IV, no recovery.

**Table 2:** Vestibular function tests of unilateral LSCC malformation patients with SSNHL.

Patient No.	Vertigo	Caloric test	cVEMP	oVEMP	vHIT	VAT
1	No	N	N	N	N	AN
2	No	N	RA	N	N	N
3	No	LA	BA	BA	N	N
4#	Yes	LA	LA	LA	LA	N
5	Yes	LA	N	BA	N	AN
6	No	RA	LA	N	N	N
7#	No	RA	BA	RA	RA	AN
8	Yes	N	LA	N	N	N

N, normal; AN, abnormal; LA, left abnormality; RA, right abnormality; BA, bilateral abnormality; #, there was dizziness before the onset of SSNHL.

**Table 3:** Clinical characteristics of bilateral LSCC malformation patients with SSNHL.

Patient No.	Sex	Age	Combined vestibular malformation	Side of SSNHL	Degree of hearing loss	Efficacy of treatment
9	M	64	L: No	L	L: Profound	II
			R: No		R: Mild	
10	M	56	L: No	R	L: Normal	IV
			R: No		R: Mild	
11	M	41	L: No	L	L: Moderate	IV
			R: No		R: Mild	
12	M	19	L: Yes	B	L: Severe	IV
			R: Yes		R: Mild	IV
13	M	35	L: Yes	L	L: Severe	II
			R: Yes		R: Normal	
14	M	42	L: Yes	R	L: Normal	II
			R: Yes		R: Severe	

M, male; F, female; L, left; R, right; B, bilateral side; I, complete recovery; II, marked recovery; III, slight recovery; IV, no recovery.

**Table 4:** Vestibular function tests of bilateral LSCC malformation patients with SSNHL.

Patient No.	Vertigo	Caloric test	cVEMP	oVEMP	vHIT	VAT
9	No	LA	N	LA	LA	AN
10	No	BA	BA	N	BA	AN
11	No	LA	N	N	N	AN
12	Yes	N	BA	LA	RA	AN
13	No	LA	N	LA	N	AN
14	Yes	RA	N	BA	RA	AN

N, normal; LA, left abnormality; RA, right abnormality; BA, bilateral abnormality; AN，abnormality.

In terms of hearing loss, of the eight patients with unilateral LSCC malformation, three (37.5%) cases were ipsilateral and five (62.5%) were contralateral. Among the six patients with bilateral LSCC malformation, one had bilateral SSNHL and the rest had unilateral SSNHL. Among the 15 ears with hearing loss, the hearing loss was mild in three ears (20.0%), moderate in two (13.3%), severe in eight (53.3%), and profound in two (13.3%); 12 ears (80.0%) were of the flat-type, one (6.7%) was of a high-frequency type, and two (13.3%) were of the total hearing loss type. We selected 165 patients as the control group. Among 165 matched patients admitted to the hospital during the same period, 88 (53.3%) had flat-type hearing loss and 77 (46.7%) non-flat-type, with a significant difference between these two groups (*p* < 0.05). Among these matched patients, 30 (18.2%) had mild hearing loss, 21 (12.7%) had moderate hearing loss, 91 (55.2%) had severe hearing loss and 23 (13.9%) had profound hearing loss, with no significant difference between these two groups (*p* > 0.05). After treatment, among the 15 treated ears, two (13.3%) showed complete recovery, three (20.0%) showed marked recovery, one (6.7%) showed a slight recovery, and nine (60.0%) had no recovery; the total effective rate was 40.0%. Among 88 controlled patients with SSNHL and without malformation who were hospitalized during the same period and had the flat type of hearing loss and less than 1 month of disease course, 10 (11.4%) showed complete recovery, 22 (25.0%) showed marked recovery, 28 (31.8%) showed a slight recovery, and 28 (31.8%) had no recovery. There was a statistically significant difference in the efficacy between these two groups (*p* < 0.05).

**Figure 1:**
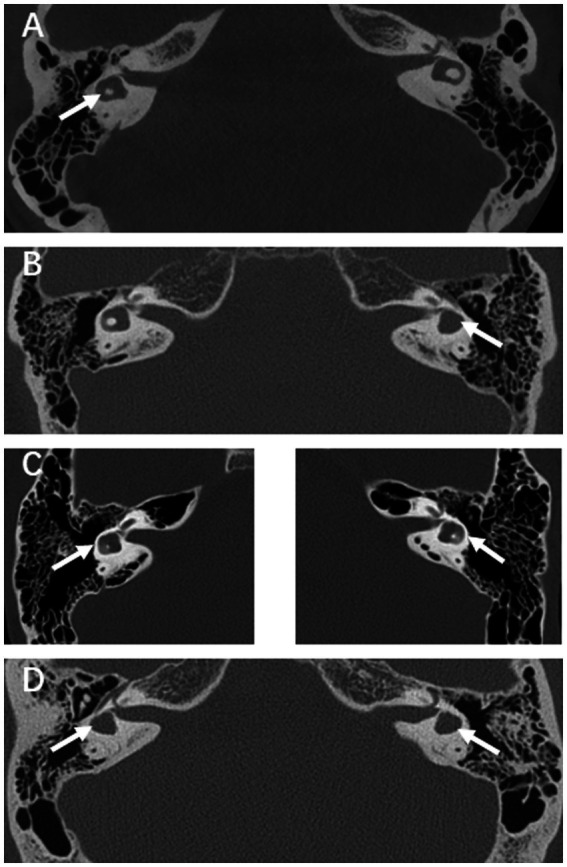
**(A)** HRCT scan of a patient with right LSCC malformation. **(B)** Example of a left LSCC malformation case with vestibular malformation. **(C)** Example of a bilateral LSCC malformations case, left and right side are placed separately. **(D)** Example of a bilateral LSCC malformations case with bilateral vestibular malformations.

Typical CT images of the LSCC and vestibular malformations are shown in Figure 1. In terms of vestibular function, patients 4 and 7 experienced dizziness before the onset of SSNHL. Dizziness was present in three (37.5%) of eight patients with unilateral LSCC malformation and two (33.3%) of six patients with bilateral LSCC malformation. Abnormal vestibular function was observed in all (100%) patients with abnormal rates of 71.4% on the caloric test, 57.1% on cVEMP, 57.1% on oVEMP, and 42.9% on vHIT (25% in unilateral LSCC and 66.7% in bilateral LSCC). The VAT anomaly rate was 64.3% (unilateral LSCC anomaly rate, 37.5%; bilateral LSCC anomaly rate, 100%; *p* < 0.05). The caloric test results showed that among eight patients with unilateral LSCC malformations, three (37.5%) were normal, one (12.5%) had an abnormal contralateral side, and four (50.0%) had an abnormal ipsilateral side. Among the six patients with bilateral LSCC malformations, one was bilateral normal, one was bilateral abnormal, and four were unilateral abnormal. Among 165 matched patients with SSNHL and without LSCC malformation during the same period, 121 (73.3%) had an abnormal vestibular function, and 44 (26.7%) had a normal vestibular function. There was a significant difference between these two groups (*p* < 0.05).

## 4. Discussion

To the best of our knowledge, this study is the first to report the clinical characteristics of SSNHL in patients with LSCC malformation.

Hearing loss or normal hearing may be associated with LSCC malformation, and the type of hearing loss may be conductive, sensorineural, or mixed (8). No correlation exists between hearing loss and isolated dysplasia in patients with LSCC (9). They may or may not have vestibular symptoms, and vestibular function tests can be normal or abnormal. Bilateral LSCC malformation with other inner ear malformations often present with profound bilateral hearing loss and vestibular diseases. Isolated unilateral LSCC malformation often presents as ipsilateral lateral semicircular canal paresis, and hearing function is usually impaired to varying degrees, but it can also be normal (10). There is also isolated LSCC malformation that may accompany sensorineural hearing loss without vestibular symptoms (11). Thus, the relationship between LSCC malformations, hearing loss, and vestibular dysfunction remains controversial.

The etiology and pathophysiological mechanisms of SSNHL have not been fully elucidated, and local and systemic factors may cause SSNHL. Thus, many factors are related to disease prognosis, which may include the characteristics of the disease, laboratory tests, and genetics (6, 12–14). Different types of SSNHL have different pathological mechanisms, curative effects, and prognostic factors (6, 15).

In this study, LSCC malformation was not accompanied by cochlear malformation, and hearing was good before onset. Patients with SSNHL mainly had flat-type hearing loss and the degree of hearing loss was severe or profound. The pathogenesis of the flat-type may be inner ear vasospasm or blood labyrinth barrier is broken, which may indicate that the inner ear vessels of patients with LSCC malformation are more likely to be abnormal (16–18). The specific mechanism needs to be further studied. It should be noted that patients with unilateral LSCC malformation may have ipsilateral and contralateral SSNHL. In this study, the ipsilateral and contralateral SSNHL incidence rates were 37.5 and 62.5%, respectively. After treatment, the disease prognosis of these patients was significantly different from that of patients with flat-type hearing loss and without LSCC malformation, indicating that LSCC malformation is a risk factor for disease prognosis.

Previous studies showed that dizziness is an uncommon symptom in patients with LSCC malformation, and even when abnormal findings are observed in vestibular function tests in patients with LSCC dysplasia, these patients may not be accompanied by dizziness (19, 20). Some studies have shown that although patients with LSCC deformity may not be accompanied by vertigo, atypical spontaneous nystagmus, such as downward pulsating nystagmus or spontaneous nystagmus changing direction, may be observed in patients with bilateral LSCC dysplasia (21). Only two patients in our study had a history of dizziness before the onset of SSNHL, and five patients experienced dizziness at SSNHL onset. This rate is similar to that of dizziness occurrence in patients without LSCC malformation (22), and patients with LSCC malformation are not more likely to have vestibular symptoms due to SSNHL. No vestibular symptoms were more likely to occur, even in patients with bilateral LSCC malformation. The absence of dizziness symptoms in patients with LSCC malformation is generally due to compensation of the central nervous system. Occurrence of dizziness may be due to incomplete compensation and insufficient residual balance function of the peripheral vestibular system. VAT examination has advantages in detecting bilateral LSCC deformities, and the abnormality rate of bilateral LSCC was significantly higher than that of unilateral LSCC. Previous studies have reported an important role of VAT in patients with vestibular migraine and decompensated Meniere’s disease (23, 24). VAT inspection should receive sufficient attention (25).

A limitation of this study is the low incidence of LSCC malformation and the small number of patients. A study with a larger number of patients is needed to obtain more reliable results. We plan to continue to treat and monitor patients in this field to obtain more reliable results.

In summary, our findings revealed that patients with SSNHL and LSCC malformation usually have flat-type and severe hearing loss. There was significant difference in the efficacy of treatment between SSNHL patients with and without LSCC malformation. Patients with LSCC malformation often have abnormal vestibular function test results, but no significant difference was noted in the vestibular symptoms between SSNHL patients with and without LSCC malformation. The findings of this study might provide a basis for diagnosis, treatment, and prognosis in clinical practice.

## Data availability statement

The original contributions presented in the study are included in the article/supplementary material, further inquiries can be directed to the corresponding author.

## Ethics statement

The studies involving human participants were reviewed and approved by the Medical Ethics Committee of the Second People’s Hospital of Shandong Province. Written informed consent for participation was not required for this study in accordance with the national legislation and the institutional requirements.

## Author contributions

LC was responsible for collecting, analyzing data, and writing the article. QD, XG, NH, XS, and HW participated in the data collection and study design. MW conceived and designed the study. All authors contributed to the article and approved the submitted version.

## Funding

This work was supported by the National Natural Science Foundation of China (No. 82271172), the Major Program of National Natural Science Foundation of China (No. 82196821), the Major Fundamental Research Program of the Natural Science Foundation of Shandong Province, China (ZR2021ZD40), and the Taishan Scholars Program of Shandong Province (No. ts20130913).

## Conflict of interest

The authors declare that the research was conducted in the absence of any commercial or financial relationships that could be construed as a potential conflict of interest.

## Publisher’s note

All claims expressed in this article are solely those of the authors and do not necessarily represent those of their affiliated organizations, or those of the publisher, the editors and the reviewers. Any product that may be evaluated in this article, or claim that may be made by its manufacturer, is not guaranteed or endorsed by the publisher.
